# Immunolocalization of platelet‐derived growth factor receptor‐β (PDGFR‐β) and pericytes in cerebral autosomal dominant arteriopathy with subcortical infarcts and leukoencephalopathy (CADASIL)

**DOI:** 10.1111/nan.12188

**Published:** 2015-04-23

**Authors:** Lucinda J.L. Craggs, Richard Fenwick, Arthur E. Oakley, Masafumi Ihara, Raj N. Kalaria

**Affiliations:** ^1^ Neurovascular Research Group, Institute of NeuroscienceNewcastle University, Campus for Ageing & VitalityNewcastle Upon TyneUK; ^2^ Department of Stroke and Cerebrovascular DiseasesNational Cerebral and Cardiovascular CenterOsakaJapan

**Keywords:** CADASIL, capillaries, dementia, pericyte, platelet‐derived growth factor receptor, vascular smooth muscle cell, vascular dementia

## Abstract

**Aims:**

Cerebral autosomal dominant arteriopathy with subcortical infarcts and leukoencephalopathy (CADASIL) is identified by aggregates of NOTCH3 extracellular domain (N3ECD) along capillaries and the deposition of granular osmiophilic material (GOM). We assessed the pattern of distribution of pericytes in relation to N3ECD deposits in cerebral microvessels of CADASIL subjects.

**Methods:**

We assessed *post mortem* brains from (*n* = 50) subjects with CADASIL, cerebral small vessel disease, and similar‐age cognitively normal and older controls. Immunohistochemical and immunofluorescent staining methods were used to study the distribution and quantify immunoreactivities of the platelet‐derived growth factor receptor‐β (PDGFR‐β) (for pericytes) and microvascular markers in the frontal cortex and white matter.

**Results:**

PDGFR‐β antibody stained cells typical of pericytes in capillaries and small arterioles in both the grey and white matter. PDGFR‐β reactive pericytes adopted ‘crescent’ morphology wrapped closely around capillary walls readily evident in cross‐sections. We noted considerable overlap between PDGFR‐β and N3ECD imunoreactivities in capillaries. Quantitative analysis of PDGFR‐β immunoreactivity revealed significant differences in PDGFR‐β %A in CADASIL compared with young controls (*P* < 0.05). PDGFR‐β %A was further positively correlated with the basement membrane marker collagen IV (*r* = 0.529, *P* = 0.009), but was not associated with GLUT‐1, the marker for endothelial cells.

**Conclusions:**

Our results suggest increased expression of PDGFR‐β immunoreactive pericytes in cerebral microvessels in CADASIL compared with similar age controls. While we cannot confirm whether PDGFR‐β‐expressing pericytes produce N3ECD and hence GOM, our findings demonstrate that up‐regulation of pericyte‐like cells is associated with microvascular changes, including loss of vascular smooth muscle cells in CADASIL.

## Introduction

Cerebral autosomal dominant arteriopathy with subcortical infarcts and leukoencephalopathy (CADASIL) is the most common hereditary stroke disorder, affecting over 600 families worldwide [1, 2, 3]. It is caused by mutations in the *NOTCH3* gene and results in aggregation of NOTCH3 extracellular domain in granular osmiophilic material (GOM) within blood vessel walls, degeneration of vascular smooth muscle cells (VSMCs) and a small vessel disease (SVD) type dementia [1, 2, 4]. We have previously shown that capillary beds are also associated with an accumulation of N3ECD deposits, which mostly represent GOM in CADASIL. It has been previously postulated that pericyte‐like cells, owing to the similar developmental lineage as VSMCs, may produce GOM. Previous electron microscopy (EM) studies have also shown that amorphous GOM is located at the cell membranes of pericyte‐like cells [5, 6, 7].

Pericytes are located abluminal to endothelial cells within the basement membrane of capillaries, pre‐capillary arterioles and post‐capillary venules [8, 9, 10]. They have multiple functions beyond contractile control of blood flow, providing paracrine signals during angiogenesis and some phagocytic activity. Pericytes are further specialized as a vital component of the blood–brain barrier (BBB) and central nervous system (CNS) capillaries have around 22–32% pericyte coverage, second in density only to capillaries of the retina [11, 12]. Pericyte processes wrap around the length of capillaries and form close contacts with endothelial cells through peg and socket junctions, gap junctions, and adhesion plaques. They are thought to contract the capillary in response to neuronal activity and during ischaemia [13]. Three major signalling pathways have been identified between endothelial and pericyte cell populations during angiogenesis: transforming growth factor‐β, angiopoietin and platelet‐derived growth factor (PDGF) [14]. In particular, PDGF subunit B expressed by endothelial cells recruits pericytes expressing PDGF receptor‐β (PDGFR‐β) to the capillary wall during vessel maturation in angiogenesis. Blockage of this signal results in fewer recruited pericytes to the vessel, causing vessel leakage, tortuosity, formation of microaneurysms and microbleeds [15]. NOTCH3 is also expressed in pericytes and perturbed NOTCH3 signalling during development results in reduced pericyte recruitment to capillaries [16]. In accord with angiogenic factors, PDGFR‐β expression is activated following ischaemic injury, where PDGFR‐β signalling surprisingly promotes transformation of VSMC to a different phenotype and loss of contractile function [17, 18]. Notch1 expression has similarly been shown to be activated by ischaemia with a similar effect to promote differentiation of VSMCs to different phenotypes [19]. In contrast, the expression of NOTCH3 is reduced following vascular injury [20]. A study exploring cultured VSMCs derived from a CADASIL patient reported that there was a negative feedback mechanism between the expression of NOTCH3 and PDGFR‐β [21], suggesting that NOTCH3 and PDGFR‐β signalling may act antagonistically to control VSMC differentiation state. Another recent study has further identified signature genes downstream of Notch activity in retinal pericytes and suggests that tight regulation of Notch signalling is crucial for pericyte survival [22]. In an effort to explore some of these mechanisms [2], we primarily aimed to localize and quantify the expression of PDGFR‐β in CADASIL in relation to N3ECD and compare against similar age relevant controls.

## Methods

### Subjects and tissues

Table 1 provides demographic details and diagnoses in the subjects. The mean ages of the CADASIL and young control subjects were not different. Available case notes and radiological reports indicated that CADASIL subjects showed extensive white matter (WM) changes consistent with small vessel disease (SVD) of the brain and met the minimum criteria for cognitive impairment used in our post‐stroke survivors study [24]. CADASIL diagnosis was confirmed by the presence of *NOTCH3* gene mutations or the presence of granular osmiophilic material (GOM) in arteries within skin biopsies [25]. None of the controls had neurological or pathological evidence for cerebrovascular disease or neurodegenerative disorder. Tissue blocks from the frontal lobe of the CADASIL subjects and age‐matched controls were collected from four sources. In addition to the Newcastle Brain Tissue Resource (NBTR), Newcastle University, Campus for Ageing and Vitality, we obtained cases from the MRC London Brain Bank for Neurodegenerative Diseases, the MRC Sudden Death Brain and Tissue Bank, University of Edinburgh and Neurology Department, Ludwig Maximilians University, Germany. Tissue from SVD subjects and older controls were obtained from the NBTR. Use of brain tissue was approved by the local research ethics committee of the Newcastle upon Tyne Hospitals NHS Foundation Trust, the NBTR committee, and the ethics committees overseeing the Brain Banks at the other respective sites.

**Table 1:** Demographic details of the cases and controls

	Young controls	CADASIL	Old controls	SVD
*N* (Total = 50)	12	11	12	10
Mean age (years) (range)	57.5	59.6	84.0	82.4
	(46–65)	(44–69)	(78–94)	(67–96)
Gender (M/F)	6/5	7/4	3/9	4/6
Age at onset (years)		46		N/A
Duration of disease		10.8 years		7–9 years
Main causes of death/notable clinical features	Heart attack, cancer, renal failure, infection	Broncho‐pneumonia, stroke/CADASIL	Heart failure, cancer, ischaemic bowel infection	Vascular dementia, heart failure, cancer, GI bleed, sudden death
Neurofibrillary Braak Stage	0	0	0–4	1–4
Mode WM Score* (range)	1	3	2	3
	(1–2)	(2–3)	(1–3)	(1–3)
PMD (h)	9–48 h	12–42	9–48 h	11–96
Fixation (months)	2–19	1–19	2–68	1–5

*WM Score; white matter pathology score assessed using scale from [23].

Length of fixation ranged 1–19 months for CADASIL, young controls and SVD samples; and 2–68 months for old controls.

CADASIL, cerebral autosomal dominant arteriopathy with subcortical infarcts and leukoencephalopathy; GI, gastrointestinal; N/A, not available; PMD, *post mortem* interval; SVD, small vessel disease; WM, white matter.

### Immunohistochemistry

Formalin‐fixed paraffin‐embedded *post mortem* brain tissue from the frontal lobe, Brodmann Area 9, was serially cut into 10‐μm thickness sections and immunostained with various antibodies, including PDGFR‐β antibody (PDGFR‐β at 1:200 dilution, clone 42G12, Product #AF385, R&D Systems, Minneapolis, MN, USA), NOTCH3 extracellular domain [N3ECD, A1‐1 antibody (1:1000 dilution, a gift from A. Watanabe)], smooth muscle alpha actin (SMA), glucose transporter‐1 (GLUT‐1) and collagen IV (COL4) as described previously [26]. Specificity of the PDGFR‐β antibody was verified per manufacturer's datasheet. Upon immunoblotting of frontal cortical samples from a young and old control, 95‐year‐old male, and a CADASIL subject, we noted the molecular weight size bands of 150 kDa. α/β tubulin bands detected at 55 kDa were recognized as loading controls (data not shown). Specificities of antibodies to vascular markers were ascertained as described previously [26, 27].

### Image acquisition and analysis

Images of capillary beds or regions of interest (ROIs) within the tissue sections were captured on a Zeiss Axioplan 2.0 microscope and Image capture software (Infinity Capture V4.6.0, Lumenera Corporation, Ottawa, Ontario, Canada), taking care to avoid larger arterioles >50 μm external diameter. Immunohistochemical staining was quantified using Image Pro Plus (V.4.0; Media Cybernetics, Silver Spring, MD, USA). We assessed the percent area (%A) for each case from at least 10 ROI images representing the vascular area stained with PDGFR‐β (as PDGFR‐β %A). To test the quality of the immunoreactvities between individual sections and cases, and we ascertained the integrated optical density (IOD). There were no significant differences in IOD values between CADASIL and control samples, however. We also found that there were no relationships between the immunohistochemical staining of PDGFR‐β and length of fixation, or *post mortem* interval, between the groups. All of the histopathological analyses were performed blind to the operator.

### Immunofluorescence (IF)

Six‐μm thick formalin‐fixed paraffin‐embedded tissue sections were cut serial to the sections used for immunohistochemistry, and underwent antigen retrieval in either 3 mM EDTA ethylenediaminetetra acetic acid buffer (pH 8.0) and blocked with 10% normal horse serum/PBS ‘phosphate buffered saline’. Sections were incubated overnight at 4°C with primary antibodies to anti‐PDGFR‐β (1:200 dilution), anti‐smooth muscle alpha‐actin (SMA, 1:500, Clone 1A4, Dako, Cambridge, UK), anti‐Notch‐3 extracellular domain (N3ECD, A1‐1 at 1:10 000), glucose transporter‐1 (GLUT‐1, 1:200, PA1‐21041 Thermo Scientific, Rockford, IL, USA). Sections were washed in PBS and incubated for 45 min with one of the following secondary antibodies: goat anti‐mouse IgG conjugated DyLight 488, (1:200, product no. 35502; Thermo Scientific); goat anti‐rabbit IgG conjugated DyLight 550 (1:200, product no. 84541; Thermo Scientific); donkey anti‐goat conjugated Alexa Fluor 350 (1:500, product no. 1084434; Life Technologies, Grand Island, New York, USA); and goat anti‐mouse conjugated Alexa Fluor 650 (1:500, product no.84545; Life Technologies). Sections were then washed in PBS before mounting in Vectashield with DAPI (Vector Laboratories, Burlingame, CA, USA). Images were captured using Leica TCS SP2 (upright) and Zeiss Spinning Disk (Invert) confocal microscopes (Leica Microsystems, Wetzlar, Germany and Zeiss Microscopy, Oberkochen, Germany). *In situ* co‐localization of two antibodies was assessed using the JaCoP Plugin for Image J [45]. The Manders co‐localization coefficient used to compare the extent of co‐localization between antibodies. The overlap of green and red (co‐localization) antigens was set to be yellow.

### Sclerotic index (SI)

Ten‐μm sections were stained with H&E haematoxylin and eosin' for assessment of the SI of microvessels in the WM. Briefly, all arterioles with external diameter between 35 and 350 μm within the region of interest were imaged using a Zeiss Axioplan 2 microscope and Image capture software (Infinity Capture V4.6.0, Lumenera Corporation). SI was measured as described previously [25, 26], and SI values of greater than 0.3 are considered to be a state of mild SVD, and those greater than 0.5 are considered to be severe [29].

### Statistics

Data were analysed using SPSS (V19.0, IBM Corporation, Armonk, NY, USA); data were tested for normality using Shapiro–Wilk test. Differences between means of groups were first tested using anova and where appropriate using Tukey's *post hoc* test . Linear correlations between PDGFR‐β immunoreactivity and other vascular markers were performed using the Pearson's correlation, as described previously [26]. The points represent %A for 23 pairs of values. Significant difference between means was considered when the *P* value was less than 0.05.

## Results

### Immunohistochemical staining of PDGFR‐β within capillaries

**Figure 1:**
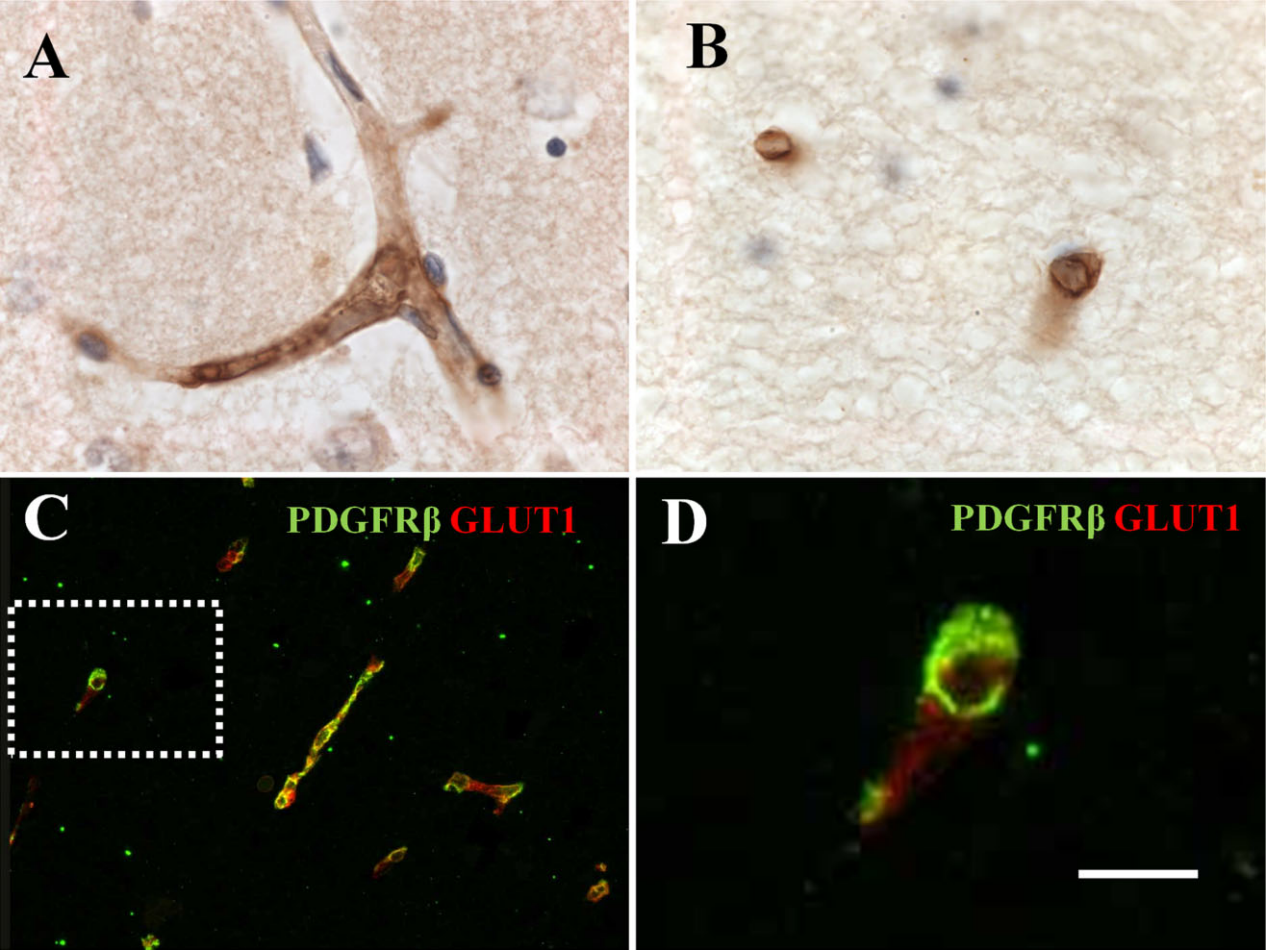
PDGFR‐β immunoreactivity in pericyte‐like cells around capillaries in the frontal white matter from a CADASIL subject. (**A**) Strong PDGFR‐β immunostaining in pericytes associated with capillaries within the cortex, with little light staining in neurons. (**B**) PDGFR‐β only stained capillaries in the white matter. (**C**) Immunofluorescent labelling with PDGFR‐β (green) and GLUT‐1 (red) antibodies. There was differential of the two markers without significant overlap along capillaries, indicating cell‐specific GLUT‐1 staining in endothelial cells, and PDGFR‐β in perivascular cells. (**D**) Higher magnification of outlined box in (**C**), demonstrating PDGFR‐β (green) immunostained pericytes, which appear as classic ‘crescent’ cell bodies with elongated processes stretching around the capillary wall. Haematoxylin counterstain in A and B. Magnification bar = 20 μm in **A**, **B** and **D**, and 50 μm in **C**. CADASIL, cerebral autosomal dominant arteriopathy with subcortical infarcts and leukoencephalopathy; PDGFR‐β, platelet‐derived growth factor receptor‐β.

PDGFR‐β antibody immunostained pericyte‐like cells associated with capillaries of the smallest internal diameters (∼6 μm) and some pre‐capillaries in both grey matter (GM) and WM (Figure 1). We also noted some PDGFR‐β immunostaining of pyramidal neurons within the cortex. Pericytes were identified by their morphology and in cross‐sections evident as typical ‘crescent’ shaped cell bodies, wrapped closely around capillary walls (Figure 1 **B**). They were also observed around the transverse length of capillaries (Figure 1 **A**,**C**). Dual IF labelling with GLUT‐1 antibodies revealed the close association between PDGFR‐β and endothelial cells, but these markers labelled different cell types (Figure 1 **C**,**D**).

**Figure 2:**
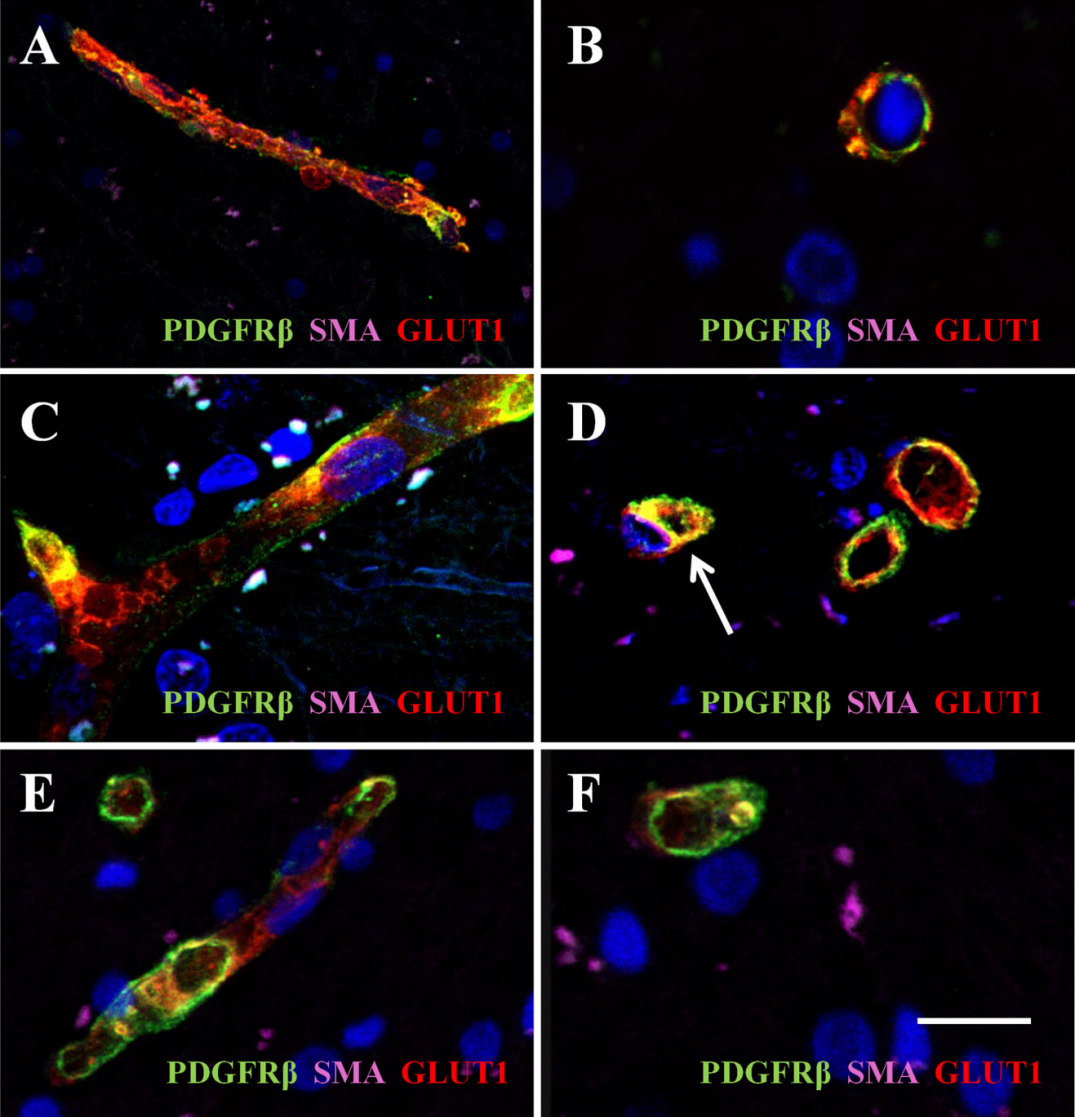
PDGFR‐β immunoreactivity in cerebral microvessels in CADASIL and cerebral SVD. Immunofluorescent labelling of PDGFR‐β (green), smooth muscle α‐actin (SMA; magenta) and GLUT‐1 (red) in capillaries in the frontal white matter, counterstained with DAPI. (**A** and **B**) Sections from a 68‐year‐old female CADASIL case with R133C mutation; (**B** and **C**), an 81‐year‐old female patient with sporadic SVD; (**E** and **F**), a 94‐year‐old nondemented female control. Severe capillary degeneration was observed in CADASIL (**A** and **B**), with GLUT‐1 expression observed in endothelial cells with blebs, compared with SVD (**C** and **D**) and control subject (**E** and **F**). There was minimal SMA immunoreactvity in pericytes (**D**, arrow) or capillaries. Magnification bar = 33.4 μm in **A**, 10.4 μm in **B**, **C** and **D**, 15 μm in **E** and 11.5 μm in **F**. CADASIL, cerebral autosomal dominant arteriopathy with subcortical infarcts and leukoencephalopathy; PDGFR‐β, platelet‐derived growth factor receptor‐β; SVD, small vessel disease.

To further differentiate and validate PDGFR‐β immunoreactivity, we performed triple labelling of capillaries with PDGFR‐β, SMA and GLUT‐1 (Figure 2). This confirmed PDGFR‐β immunoreactivity was largely associated with pericytes in capillaries in the general absence of SMA reactivity. However, we did observe both PDGFR‐β and SMA immunoreactivities in some larger capillaries with diameters greater than 10 μm (Figure 2 **D**). In CADASIL cases, there were often severely degenerating capillaries, where expression of the endothelial protein GLUT‐1 was perturbed and some perivascular cells stained positive (possibly leaked erythrocytes) for GLUT‐1 with evidence of endothelial blebbing (Figure 2 **A**,**B**). This was not frequent in SVD and less so in control.

**Figure 3:**
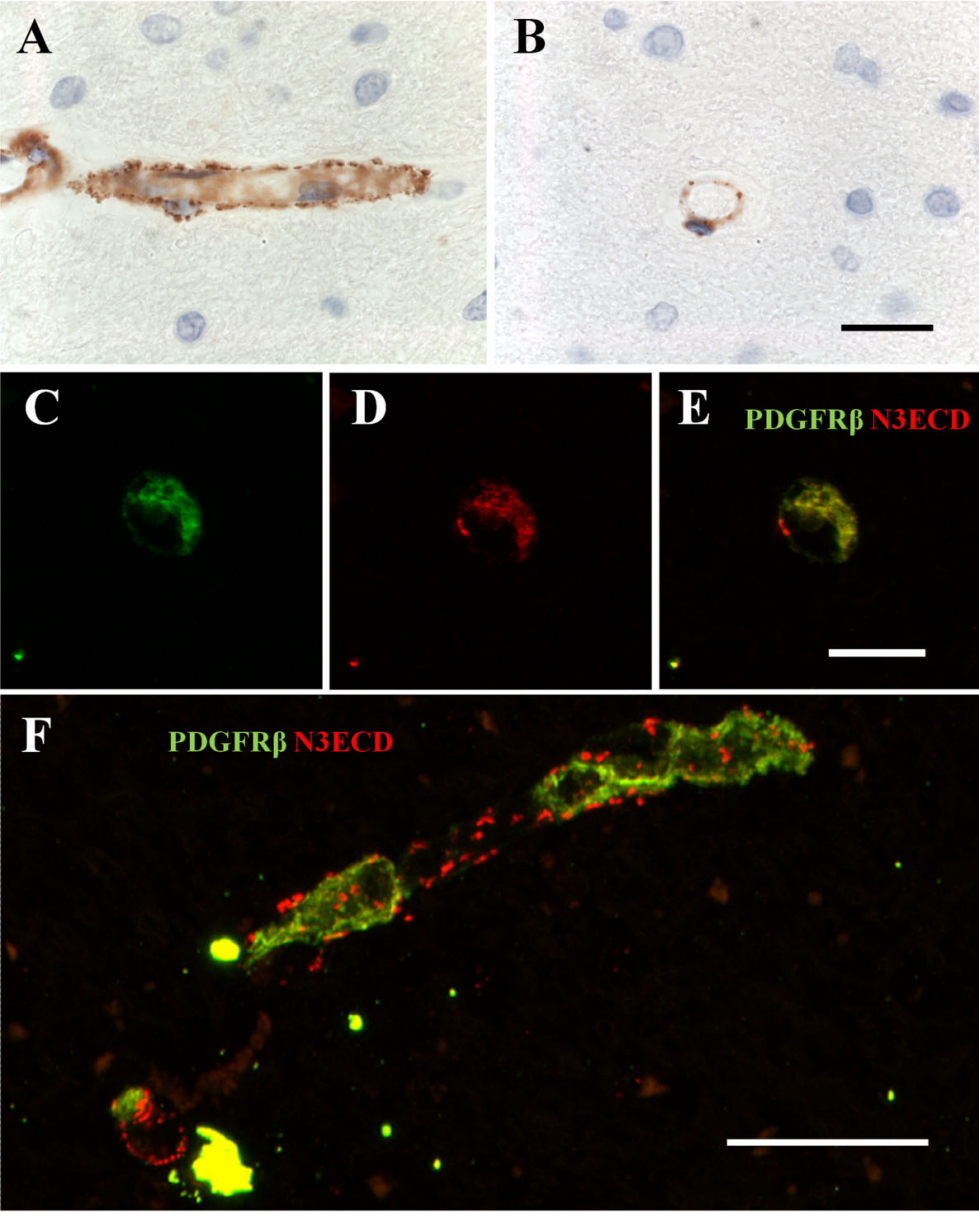
PDGFR‐β immunoreactivity in relation to N3ECD deposits, as a marker of GOM along capillaries in CADASIL. **A** and **B**, N3ECD immunostaining in micro‐deposits (GOM) around capillaries. *Post mortem* human brain tissue from a 68‐year‐old female CADASIL case with R133C mutation. (**C**) PDGFR‐β (green) immunostained pericyte in cross‐section. (**D**) N3ECD (red) accumulation around the same pericyte (as in **C**). (**E**) Composite of both PDGFR‐β (green) and NOTCH3ECD (red), demonstrating co‐localization for both antibodies around the pericyte. (**F**) Section of capillary with PDGFR‐β (green) and NOTCH3ECD (red) immunoreactivities, demonstrating abundance of N3ECD deposits (red) associated with the capillary wall. There is some overlap with pericytes within the tissue. Magnification bar = 20 μm in **A** and **B**, 11 μm in **C**–**E**, and 24 μm in **F**. CADASIL, cerebral autosomal dominant arteriopathy with subcortical infarcts and leukoencephalopathy; GOM, granular osmiophilic material; PDGFR‐β, platelet‐derived growth factor receptor‐β.

To assess the relationship between pericytes and N3ECD deposits or GOM in CADASIL, we first replicated our previous findings to show that N3ECD immunoreactivity was localized as granular material along capillaries (Figure 3). We then used dual IF labelling to assess co‐localization of PDGFR‐β positive pericytes and N3ECD deposits. We observed N3ECD‐stained granular deposits along capillary walls, indicating an abundance of N3ECD and likely GOM deposits within the capillary beds, but these did not always overlap with PDGFR‐β‐immunostained cells (Figure 3 **D**). However, upon overall screening, PDGFR‐β positive pericytes were co‐localized with N3ECD in ∼70% of the CADASIL cases. Using dual IF labelling to assess co‐localisation between PDGFR‐β positive pericytes and N3ECD deposits we found strong association between the two antibodies with the following statistics: Pearson's *r* ^2^ = 0.905; Manders' coefficients M = 0.969 for fraction of N3ECD overlapping PDGFR‐β, and M = 0.920 for fraction of PDGFR‐β overlapping N3ECD (*P* < 0.001). This indicated a close relationship between pericytes with N3ECD deposits or GOM.

### Immunohistochemical staining of PDGFR‐β within larger cerebral microvessels

**Figure 4:**
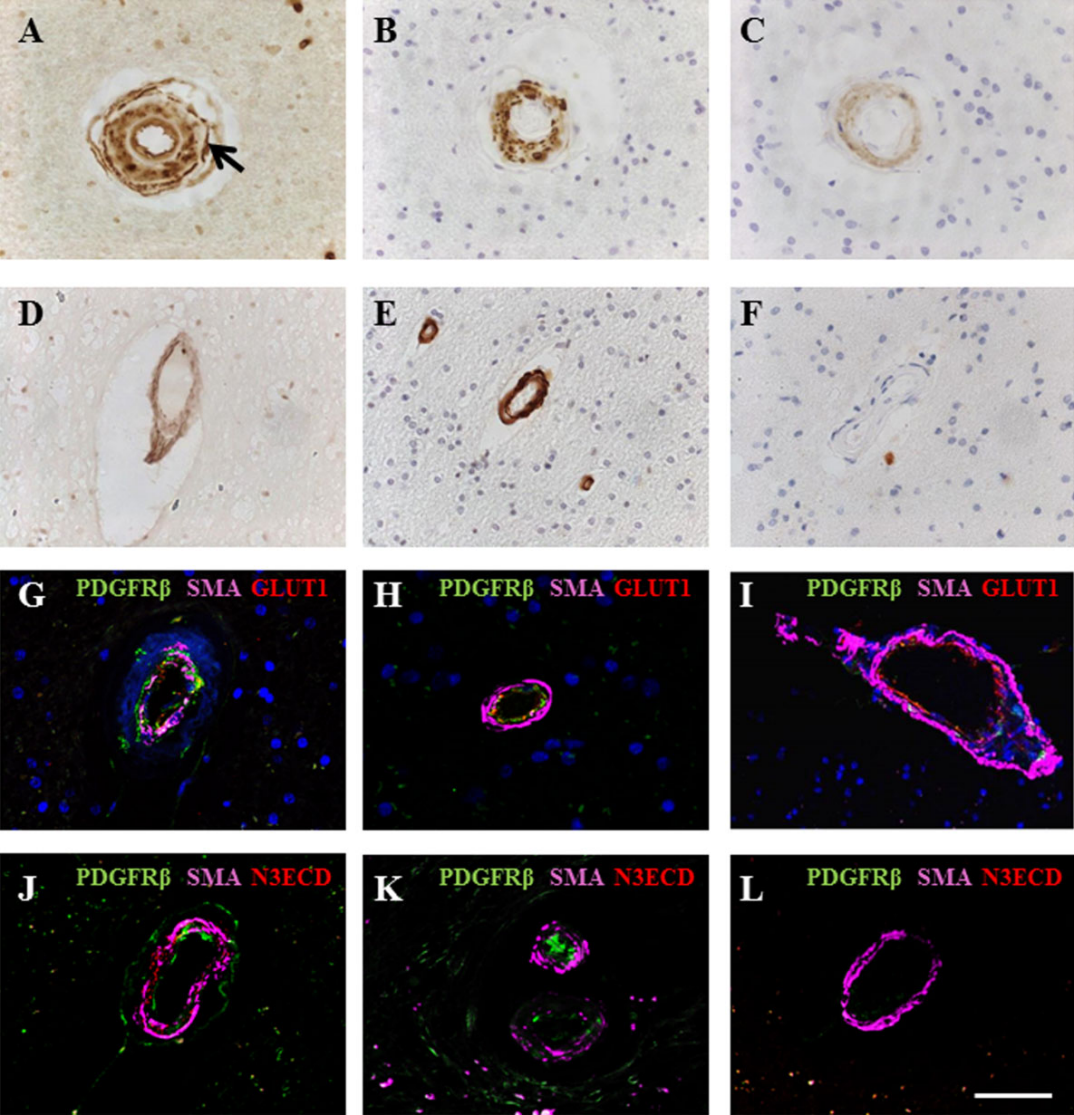
PDGFR‐β immunoreactivity associated with arterioles and cerebral microvessels in CADASIL. (**A**–**C**) Immunohistochemical staining for PDGFR‐β (**A**), SMA (**B**) and N3ECD (**C**) in a partially hyalinized arteriole in the white matter of a 44‐year‐old female CADASIL subject with R153C mutation. (**A**) PDGFR‐β reactivity was observed in the endothelial cells of arterioles and expressed abluminally to VSMC (arrow) and in the adventitia. (**B**) SMA and N3ECD reactivities were predominantly within the tunica media of the same vessel (**C**). (**D**–**F**) PDGFR‐β immunostaining in 49‐year‐old cognitively normal control female, indicating diffuse PDGFR‐β staining within arteriolar walls (**D**). SMA staining revealed intact vascular smooth muscle cells (**E**) without presence of N3ECD aggregates (**F**). (**G**–**I**) Immunofluorescent labelling of PDGFR‐β (green), SMA (magenta) and GLUT‐1 (red) in arterioles of the frontal white matter, with DAPI counterstain. (**G**) Vascular smooth muscle cell (demonstrated by SMA, magenta) degeneration in arterioles in a 68‐year‐old female CADASIL case with R133C mutation, with increased PDGFR‐β (green) expression within the vessel wall. (**H**) Some PDGFR‐β (green) reactivity in the endothelial cell layer of arteriole from an 81‐year‐old female patient with sporadic SVD with mild VSMC degeneration compared with CADASIL (cf. **D**). (**I**) PDGFR‐β (green) expression was absent in an arteriole in a 94‐year‐old nondemented female control, although GLUT‐1 (red) and SMA (magenta) appeared normal. (**J**–**L**) Immunofluorescent labelling of PDGFR‐β (green), SMA (magenta) and N3ECD (red) in arterioles of white matter, confirming that N3ECD only accumulated in the tunica media in CADASIL (**J**), although PDGFR‐β (green) immunoreactivity was apparent in both arterioles from CADASIL (**J**) and SVD cases (**K**). (**L**) GLUT‐1 immunostaining in endothelial cells of arteriole of a control subject, whereas PDGFR‐β reactivity was meagre, if any. Scale bar = 50 μm in **A**–F, 40 μm in **G**, 27.5 μm in **H**, 47.5 μm in **I**, 52 μm in **J**, 57 μm in **K** and 49.5 μm in **L**. CADASIL, cerebral autosomal dominant arteriopathy with subcortical infarcts and leukoencephalopathy; PDGFR‐β, platelet‐derived growth factor receptor‐β; SMA, smooth muscle alpha actin; SVD, small vessel disease; VSMC, vascular smooth muscle cell.

**Figure 5:**
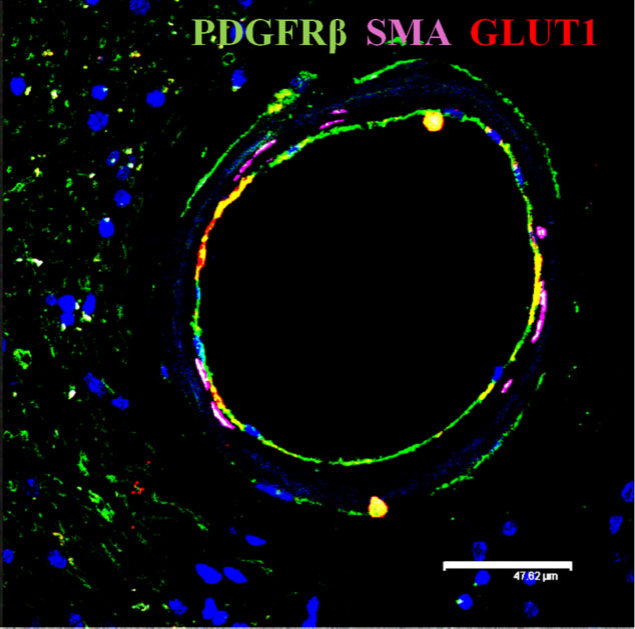
Immunofluorescent labelling of PDGFR‐β (green), SMA (magenta) and GLUT‐1 (red) in an arteriole within the frontal white matter of a 68‐year‐old female CADASIL subject with R133C mutation. Expression of GLUT‐1 (red) by endothelial cells was markedly diminished in this arteriole. However, there was marked PDGFR‐β (green) reactivity. Scale bar is 47.62 μm. CADASIL, cerebral autosomal dominant arteriopathy with subcortical infarcts and leukoencephalopathy; PDGFR‐β, platelet‐derived growth factor receptor‐β; SMA, smooth muscle alpha actin.

PDGFR‐β antibody staining was also observed in endothelial cell layers, in cells located within the tunica media and more diffusely in the adventitia of small arterioles (internal diameter approximately 30 μm) in CADASIL, SVD cases and less so in controls (Figure 4). However, diffuse and granular PDGFR‐β staining within the intima was observed in CADASIL subjects where vessels had undergone hyalinosis (SI >0.5), but to a lesser extent in SVD (Figure 4). PDGFR‐β immunostained smaller arterioles more frequently, concomitant with degenerated smooth muscle cells and N3ECD deposits (Figure 4 **B**,**C**). Using multiple IF staining for PDGFR‐β, SMA and GLUT‐1, we assessed the distribution of the markers in CADASIL, SVD and controls. We observed that in both CADASIL and SVD arterioles, there was up‐regulation or enhanced PDGFR‐β immunoreactivity in endothelial cells compared with controls with positive GLUT‐1 but little PDGFR‐β immunostaining (Figure 4 **G**–**I**). In some large arterioles in CADASIL cases, there was near lack of GLUT‐1 reactivity, but strong PDGFR‐β reactivity (Figure 5). We also observed PDGFR‐β staining within the tunica media and adventitia of arterioles more frequently in CADASIL compared with SVD, and found concomitant immunostaining for N3ECD alongside degeneration of VSMC in CADASIL arterioles, a change which was not observed in SVD or controls (Figure 4 **J**–**L**).

### Quantitative analysis of PDGFR‐β and the relationship with vascular markers

**Figure 6:**
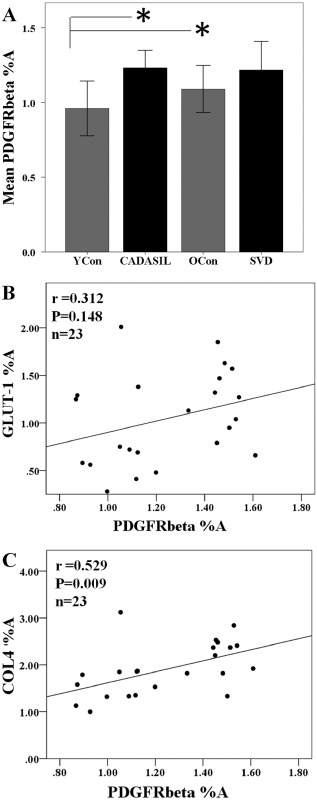
Quantification of PDGFR‐β immunoreactivity in the frontal white matter in CADASIL. (**A**) Quantitative analysis of PDGFR‐β percent area (%A) revealed a trend for differences between the groups (anova, *P* = 0.084, *n* = 6–7 samples per group). However, *post hoc* tests revealed significant differences between CADASIL subjects and young controls, and the old control group compared with young controls (Tukey's test, *P < 0.05). (**B**) Lack of relationship between PDGFR‐β %A and GLUT‐1 immunoreactivity (*r* = 0.312, *P* = 0.148). (**C**) PDGFR‐β %A was significantly correlated with the basement membrane marker COL4 (*r* = 0.529, *P* = 0.009). CADASIL, cerebral autosomal dominant arteriopathy with subcortical infarcts and leukoencephalopathy; PDGFR‐β, platelet‐derived growth factor receptor‐β.

Quantitative analysis in capillaries in the frontal WM immunostained for PDGFR‐β revealed a trend for differences in the %A immunoreactivity between groups (anova, *P* = 0.084). However, *post hoc* tests revealed significant differences between CADASIL and young control, and between old and young controls (Tukey's test, *P* < 0.05; Figure 6 **A**).

To assess if PDGFR‐β immunostaining was related to changes in vascular cell markers, we correlated the PDGFR‐β %A determined in the frontal WM against the endothelial cell marker, GLUT‐1 and the basement membrane marker, COL4. We found no association between the %A immunostaining of PDGFR‐β and GLUT‐1 (Pearson's *r* = 0.312, *P* = 0.148, *n* = 23, Figure 6 **B**). However, we observed a significant correlation between %A immunostaining of PDGFR‐β and COL4 (Pearson's *r* = 0.529, *P* = 0.009, *n* = 23, Figure 6 **C**). We further calculated the PDGFR‐β %A: COL4 %A ratio of the CADASIL cases and observed that there were no significant differences between the young control and CADASIL groups, indicating lack of changes in overall PDGFR‐β immunoreacvity per unit amount of COL4 or basement membrane protein (*P* > 0.05). To also relate whether arteriolar sclerosis in general had any impact on capillary density, we correlated PDGFR‐β immunoreactivity as %A with SI and found no relationship between %A staining of PDGFR‐β and SI (data not shown).

## Discussion

Using immunohistochemical methods, we localized PDGFR‐β immunoreactivity in brains of subjects with CADASIL and SVD. Concentrating on the WM, we confirmed that PDGFR‐β positive cells are pericyte‐like cells frequently found around capillaries. These cells are also associated with N3ECD immunoreactivity, which is a reliable marker of GOM in CADASIL [4]. From these static studies, it is not clear whether pericytes produce or uptake N3ECD or GOM as a measure for perivascular clearance [30]. However, our findings are consistent with the postulation that pericytes, identified by electron microscopy, are associated with GOM [5, 6, 7] and by extension N3ECD deposits.

We also showed that there is an increase in PDGFR‐β immunoreactivity associated with pericytes in CADASIL compared with similar age controls, with a similar trend in SVD compared with respective older controls. These results suggest changes in the capillary population of PDGFR‐β‐expressing pericytes in the WM in CADASIL. The findings further suggest that pericyte coverage of capillaries is robust in CADASIL despite the presence of a significant burden of N3ECD deposits or GOM in their proximity. This possibly indicates that the integrity of the BBB is not completely diminished because it has been previously shown that the loss of pericytes in PDGF‐null mice impairs the barrier [9]. We also observed that PDGFR‐β immunoreactivity was associated with COL4 immunoreactivity, but not GLUT1 in capillaries. This suggests that immunoreactivity to PDGFR‐β or density of pericytes was not changed in relation to changes in COL4 reactivity, but does confirm the known relationship between pericytes and capillary basement membranes [9]. The lack of a significant relationship between PDGFR‐β and GLUT‐1 may partly reflect abnormalities in endothelial cells, which are also in close proximity to pericytes [9].

PDGFR‐β is known to be up‐regulated in response to hypoxia [19, 31] and its expression can be induced in different cell types under different conditions [32]. A recent reported a significant reduction in the number of pericytes following acute stroke, followed by proliferation of PDGFR‐β+ cells of neurovascular origin during scar formation [31]. However, this study utilized a model of acute large artery occlusion which produces a large ischaemic lesion [31], a process not common to CADASIL or SVD [2, 3]. Instead, CADASIL is known to undergo slow insidious progression with multiple small strokes (and transient ischaemic attacks), resulting in microinfarcts and an overall loss of axonal myelin without related WM changes [2, 3, 26]. We therefore postulate a differential involvement of PDGFR‐β‐expressing pericytes in CADASIL and possibly SVD, where a chronic hypoxic state [33] or low levels of transient hypoxia may up‐regulate various factors such as bone morphogenic protein‐4 (Maiko Uemura and Masafumi Ihara, pers. comm.), which co‐localizes with PDGFR‐β and may in turn affect its expression in pericytes. This, however, does not result in large‐scale increases in PDGFR‐β‐cells migrating into the parenchyma to form scar tissue.

PDGF is also expressed by endothelial cells during angiogenesis to attract PDGFR‐β‐expressing pericytes to capillary walls, where PDGF acts as a downstream of angiogengic signal, which in turn increases in response to hypoxic signals [15, 34]. Further to involvement in pericyte recruitment in angiogenesis, PDGF expression interacts with Notch signalling, where both Notch1 and Notch3 ligands promoted PDGFR‐β expression in cultured VSMC through independent mechanisms, but both receptors appear to instigate increased functional PDGFR‐β expression [21]. This mechanism may serve to transiently attenuate VSMC phenotype in response to extracellular cues. A muted response of PDGFR‐β up‐regulation in cultured VSMC from a CADASIL patient with the R133C *NOTCH3* mutation [21], a version of NOTCH3 receptor thought to be hypomorphic, although the functional phenotype of CADASIL associated *NOTCH3* mutations, is still under debate [35, 36]. These findings may explain the observed increase in PDGFR‐β staining in capillaries of CADASIL, where dysfunctional NOTCH3 mutant proteins present in CADASIL result in an exacerbated response of PDGF and PDGFR‐β expression in states of hypoxia to promote angiogenesis.

We observed PDGFR‐β reactivity in endothelial cells of small arterioles, where the staining was more intense frequently in CADASIL. We further observed (qualitative) diffuse PDGFR‐β immunostaining within the vessel wall, specifically in the small arterioles, which had undergone sclerosis of the vessel wall. PDGF signalling has been associated with other vascular pathologies, such as atherosclerosis [37, 38], kidney fibrosis [39], lung fibrosis [32], pulmonary arterial hypertension (PAH) [40, 41] and diabetes [42]. Some of these vascular disorders exhibit similar pathophysiology to those observed in arteriolosclerosis and hyalinosis of arterioles commonly observed in dementing cerebrovascular disorders, where vessels exhibit significant vessel wall thickening, increased collagen deposition and changes in VSMC morphology [26]. Similarly, in atherosclerosis and arterial restenosis, VSMCs de‐differentiate from their contractile state towards a less specialized cell type, switching to a synthetic phenotype and migrating into the intima where they undergo proliferation, a mechanism regulated by PDGFR‐β signalling [32, 37, 42]. We previously reported that SVD and CADASIL cases exhibit increased arteriolosclerosis in the frontal WM [26]. Taken together, our results suggest that increased PDGFR‐β in endothelial cells of arterioles may be linked to degeneration of VSMC and vessel wall hyalinosis.

Our results suggest that there is a similar mechanism responsible for the vessel sclerosis observed in small vessel diseases of the brain as that which has been reported in other systemic disorders, where an increase in PDGF signalling is initiated by a primary vascular disorder such as hypertension or diabetes. This PDGF signal results in a phenotype conversion of VSMCs from contractile to a synthetic phenotype, which would function to reduce the contractile ability of the blood vessel and therefore a loss of autoregulation of cerebral blood flow. Once the VSMCs begin to dedifferentiate, the control of local blood flow may be attenuated, resulting in further states of hypoxia, which in turn up‐regulates the Notch and PDGF pathways, and the vessel is further impeded in a vicious cycle. Although the consequences of CADASIL‐causing mutations are debated [35, 36], *NOTCH3* mutations might be considered the primary factor in the disease process, and the similarities between CADASIL and SVD could be explained by *NOTCH3* polymorphisms associated with late‐onset SVD [35, 43], where presence of a hypomorphic NOTCH3 phenotype may render a patient more vulnerable to ischaemic processes occurring as a result of other environmental damage to blood vessel walls, such as hypertension.

Our study is not without limitations. The availability of a larger pool of brain samples would have permitted more in‐depth characterization of the PDGFR‐β‐expressing pericytes in the WM regions. The availability of more specific markers of pericytes would also have been useful to verify our findings. However, our initial study is sufficiently novel to suggest that the persistent hypoxic state within the deep WM [44] may reflect activation of capillary‐associated pericytes in CADASIL. The significance of these findings is important for both CADASIL and SVD subjects, where agents, which modulate PDGF signalling, may provide therapeutic strategies to reduce the morbidity in cerebral SVDs.

## Sources of funding

Our work is supported by grants from the UK Medical Research Council (MRC, G0500247), Newcastle Centre for Brain Ageing and Vitality (BBSRC, EPSRC, ESRC and MRC, LLHW) and Alzheimer's Research (ARUK). Tissue for this study was collected by the Newcastle Brain Tissue Resource, which is funded in part by a grant from the UK MRC (G0400074), by the Newcastle NIHR Biomedical Research Centre in Ageing and Age Related Diseases award to the Newcastle upon Tyne Hospitals NHS Foundation Trust, and by a grant from the Alzheimer's Society and ART as part of the Brains for Dementia Research Project.

## Author contributions

Lucinda J. L. Craggs: analysis and interpretation of data, acquisition of the microvascular results, drafting and revising the manuscript at various stages of preparation.

Richard Fenwick: analysis and acquisition of the original SI data.

Arthur E. Oakley: analysis/interpretation/acquisition of data and technical advice on imaging.

Masafumi Ihara: editing and revising the manuscript, and interpretation of the analysis.

Raj N. Kalaria: drafting, revising the manuscript and interpretation of data, diagnosing the cases and obtaining funding.

## Disclosures

There are no disclosures relating to this paper.
